# The thermogenic actions of natriuretic peptide in brown adipocytes: The direct measurement of the intracellular temperature using a fluorescent thermoprobe

**DOI:** 10.1038/s41598-017-13563-1

**Published:** 2017-10-11

**Authors:** Haruka Kimura, Tomohisa Nagoshi, Akira Yoshii, Yusuke Kashiwagi, Yoshiro Tanaka, Keiichi Ito, Takuya Yoshino, Toshikazu D. Tanaka, Michihiro Yoshimura

**Affiliations:** Division of Cardiology, Department of Internal Medicine, The Jikei University School of Medicine, Tokyo, Japan

## Abstract

In addition to the various effects of natriuretic peptides (NPs) on cardiovascular systems, increasing attention is being paid to the possibility that NPs induce adipose tissue browning and activate thermogenic program. We herein established a direct intracellular temperature measurement system using a fluorescent thermoprobe and investigated the thermogenic effects of A-type NP (ANP) on brown adipocytes. The thermoprobe was successfully introduced into rat brown adipocytes, and the temperature dependent change in fluorescence intensity ratio was measured using a fluorescence microscope. After one-hour incubation with ANP, the degree of the change in fluorescence intensity ratio was significantly higher in ANP-treated (P < 0.01) adipocytes compared to untreated controls. The ANP treatment increased uncoupling protein-1 (UCP1) mRNA levels, which is one of the markers of thermogenesis in adipocytes, while the intracellular ATP content was not changed, indicating mitochondrial uncoupled respiration. Intriguingly, these thermogenic actions of ANP were more prominent when brown adipocytes were incubated at 35 °C than at 37 °C. Moreover, the increase in the intracellular temperature and the expression of UCP1 induced by ANP were cancelled by p38MAPK inhibition. Taken together, this study directly demonstrated the thermogenic actions of ANP in brown adipocytes through the use of a novel method of intracellular temperature measurement.

## Introduction

The various effects of natriuretic peptides (NPs) on the cardiovascular system play a key role in the pathophysiology of heart failure. A-type natriuretic peptide (ANP) and B-type natriuretic peptide (BNP), both of which are hormones that are produced in the heart, regulate blood pressure and fluid homeostasis through vasodilatory and diuretic actions, and improve cardiac remodeling, mainly through the inhibition of the renin-angiotensin-aldosterone and the sympathetic nervous systems^1–4^. Although NPs classically act on the renal and cardiovascular systems, recent studies have indicated that NPs also coordinate inter-organ metabolic crosstalk largely to adipose tissues, in which NP receptors are expressed^5–11^.

In contrast to white adipocytes, which primarily function as an energy storage depot, brown adipocytes promote energy utilization and generate heat using metabolic fuel^8–10,12,13^. Uncoupling protein 1 (UCP1) is specifically expressed in brown adipose tissue (BAT), and enables mitochondrial uncoupled respiration, rather than ATP production, allowing for the dissipation of nutritional energy as heat^12–14^. There is a well-established physiological pathway that controls lipolysis through the activation of the β-adrenergic receptors (β-ARs), and the β-AR-cAMP pathway subsequently activates the process of adaptive thermogenesis via UCP1^9,14,15^. Emerging evidence suggests that NPs also stimulate triglyceride lipolysis, promote the uncoupling of mitochondrial respiration, and activate thermogenesis^7–10,15,16^. After the binding of NPs to their receptor, NPR-A, cGMP is produced, which activates cGMP-dependent protein kinase (PKG), and PKG-mediated phosphorylation triggers lipolysis. Meanwhile, PKG also activates p38 mitogen-activated protein kinase (MAPK), leading to the induction of the BAT thermogenic program through the PGC1α-UCP1 pathway^8,9,15^. In fact, mRNA levels of ANP and BNP in the heart, as well as the plasma BNP levels, are elevated in mice exposed to cold environments, which supports the idea that NPs, in parallel with the sympathetic input described above, could induce the activation of the adipose tissue thermogenic program in response to cold stimulus^15^. Although increasing attention is being paid to the possibility that NPs may induce adipose tissue browning and activate the thermogenic program, no reports have demonstrated the thermogenic actions of NPs in adipose tissue by directly measuring the intracellular temperature.

Methods have been developed to measure the intracellular temperature of living cells using a fluorescent polymeric thermometer^17,18^. The fluorescent polymeric thermometer diffuses throughout the cells and provides information about the distribution of the intracellular temperature based on the fluorescence ratio. Recently, a novel thermoprobe, which can be delivered into cells without microinjection, has become available. We established a system that allows for the direct measurement of the intracellular temperature using this fluorescent thermoprobe under a fluorescence microscope. In order to investigate the thermogenic effects of NPs on BAT more directly, we measured the intracellular temperature of brown adipocytes after ANP stimulation using this newly established measurement system.

## Results

### The thermoprobe successfully detected the intracellular temperature of rat brown and white adipocytes

**Figure 1:**
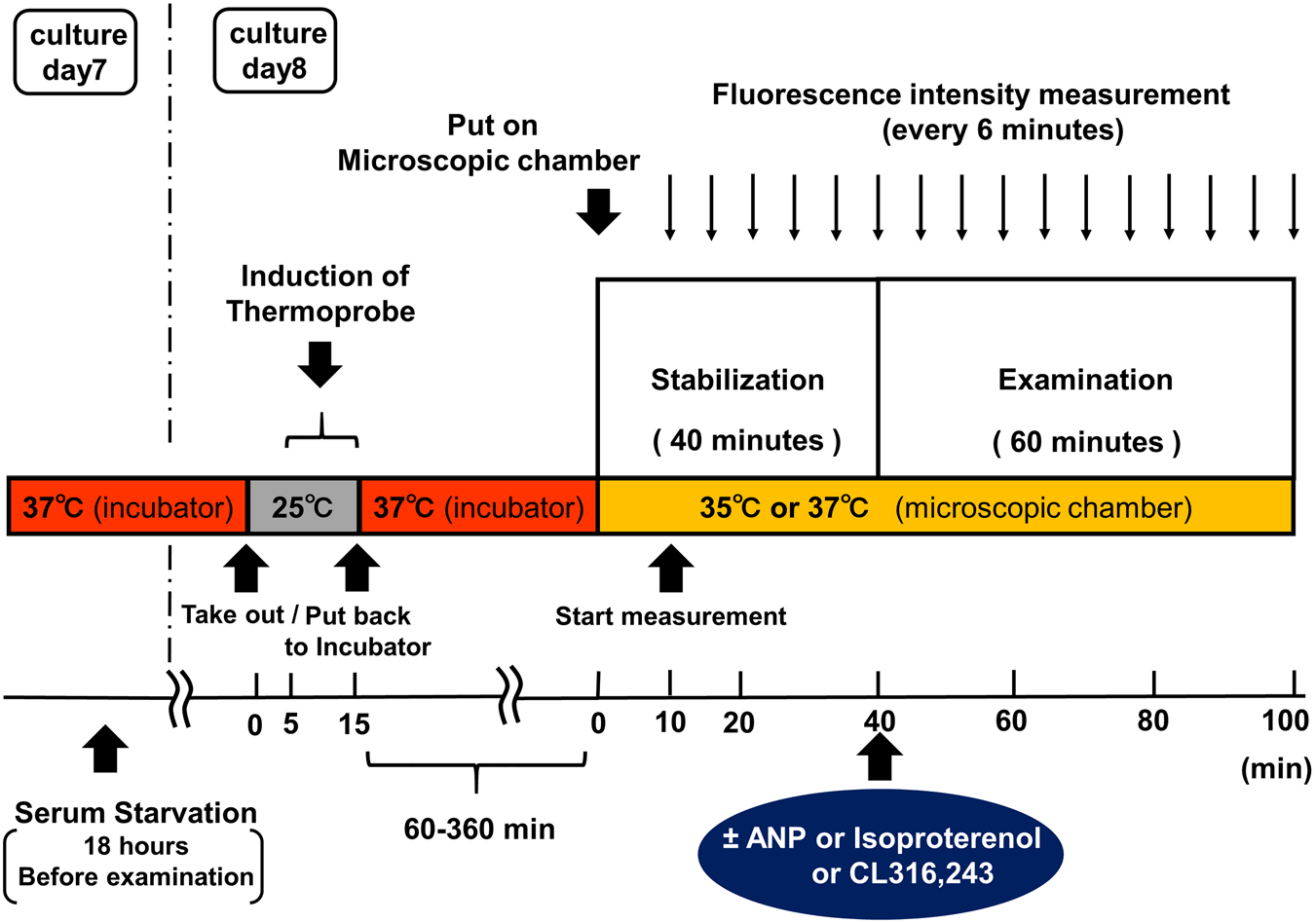
The experimental protocol showing the duration and the time course of the intracellular temperature measurement. Brown and white adipocytes treated with the cellular thermoprobe were incubated at 37 °C, and were placed in the microscopic chamber at 35 °C or 37 °C. The fluorescence intensity and ratio were measured every 6 minutes. After stabilization for 40 minutes, atrial natriuretic peptide (ANP), isoproterenol, or CL316,243 was added to the indicated sample dish.

**Figure 2:**
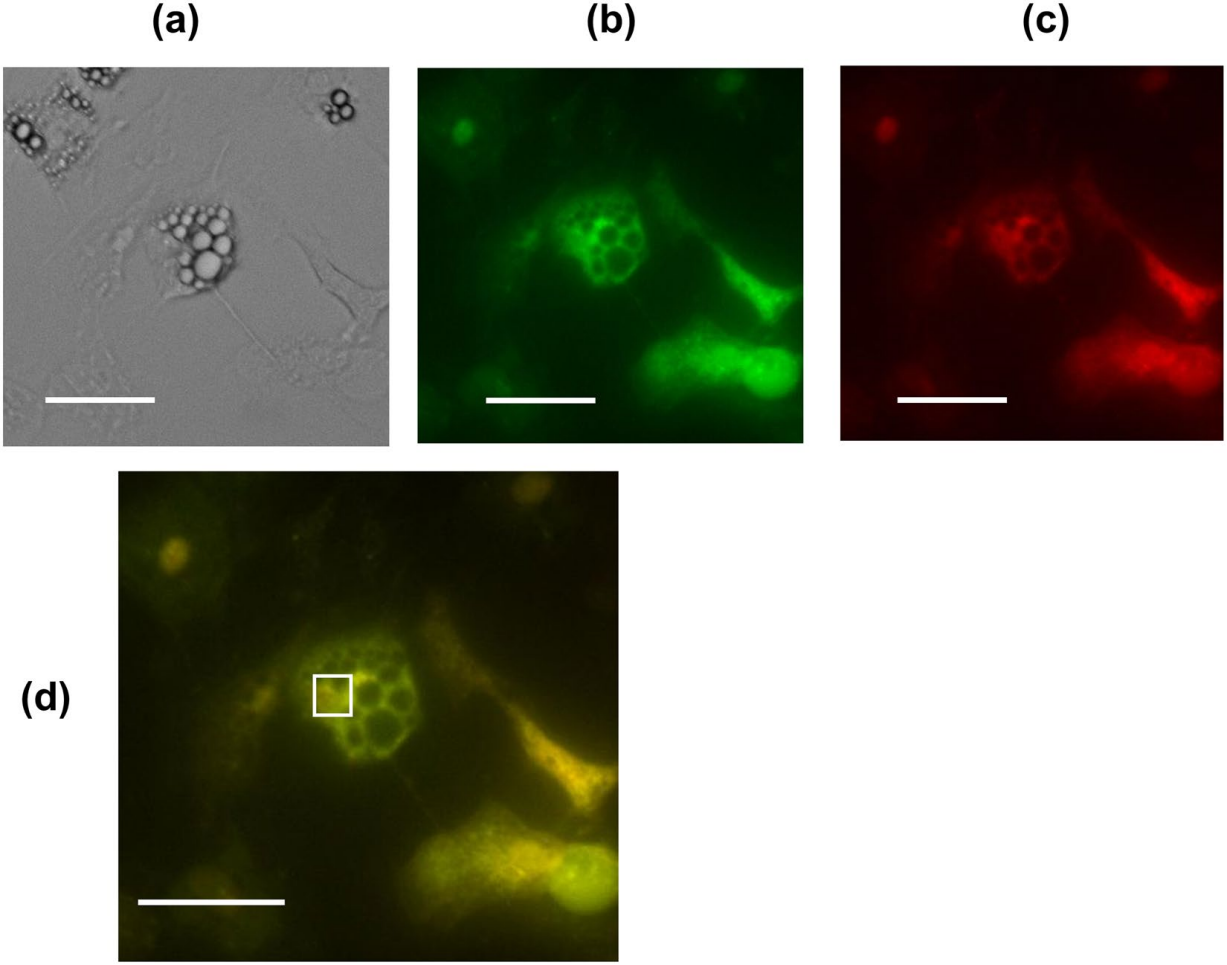
Representative microscopic images of rat brown adipocytes treated with the fluorescent polymeric thermometer. A differential interference contrast image (**a**), a fluorescence image (490 nm excitation, 525 nm emission), (**b**) and a fluorescence image (490 nm excitation, 605 nm emission) (**c**) of the cellular thermoprobe in rat brown adipocytes on day 8. A merged image of (**b**) and (**c**) with the sampling square of the measurement are also shown in (**d**). Scale bar: 40 µm.

**Figure 3:**
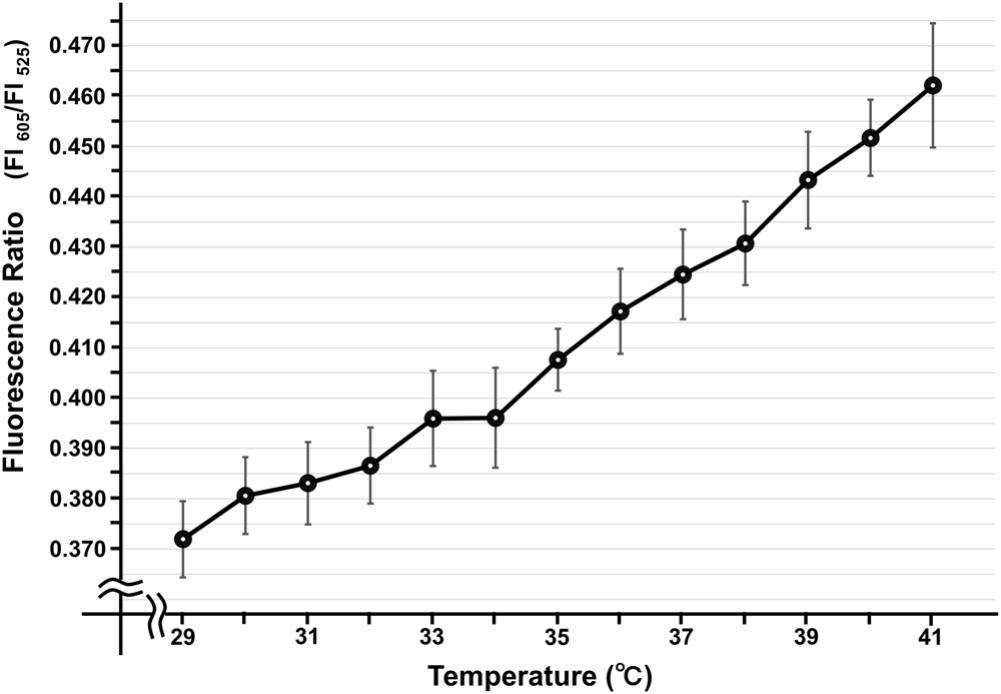
The calibration curve of the fluorescent polymeric thermometer in rat brown adipocytes. The responses of the fluorescence ratio (605 nm/525 nm) were analyzed (n = 4). The data indicate the mean ± SEM.

The protocol of the present study is shown in Fig. 1, and is described in detail in the Methods section. Representative microscopic images of the rat brown and white adipocytes are shown in Fig. 2 and Supplementary Fig. S1, respectively. Multiple lipid droplets were detected inside the cultured cells (day 8), indicating that these cells were adipocytes (Fig. 2a and Supplementary Fig. S1a). We confirmed that the fluorescent polymeric thermometers were successfully introduced into living brown and white adipocytes in two different settings of excitation/emission (Figs. 2b,c, Supplementary Fig. S1b and S1c). To measure the intracellular temperature, cellular cytoplasm was selected in a merged fluorescence image from two different values of excitation/emission (Fig. 2d and Supplementary Fig. S1d). To confirm that the thermoprobe used in the current study could detect the subtle changes in the intracellular temperature, a calibration curve was prepared (Fig. 3 and Supplementary Fig. S2). The temperature-dependent change in the fluorescence intensity ratio of the cellular thermoprobe was observed at 605 to 525 nm (FI 605/FI 525), indicating that the fluorescence ratio measured by this polymetric thermometer was an appropriate marker for assessing the temperature in living cells.

### The warming effects by the thermogenic actions of ANP in brown adipocytes

**Figure 4:**
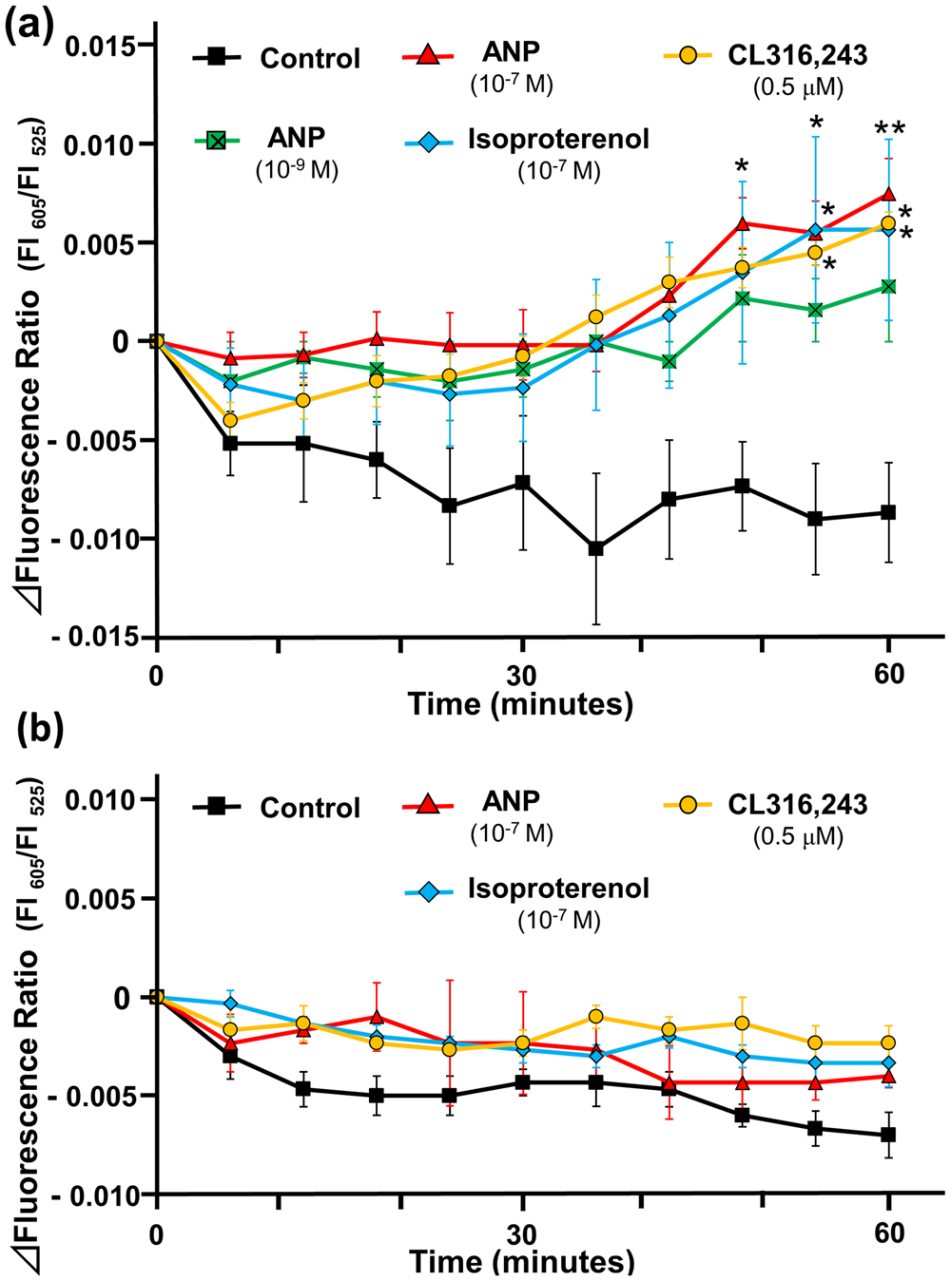
The profile of the intracellular temperature change in rat brown adipocytes incubated with ANP. The intracellular temperature was indicated by the fluorescence ratio (605 nm/525 nm). The changes of the fluorescence ratio in rat brown adipocytes (day 8) after treatment with ANP (10^−9^ M, n = 5; 10^−7^ M, n = 6), isoproterenol (10^−7^ M, n = 6), or CL316,243 (0.5 µM, n = 4) were recorded every 6 minutes (Control, n = 6) at 35 °C (**a**) or 37 °C (**b**). The data represent the mean ± SEM. *P < 0.03 and **P < 0.01 versus the controls at each time point (one-way ANOVA followed by Bonferroni’s multiple comparisons test).

The changes in the intracellular temperature were measured every 6 minutes for 60 minutes either at 35 °C or at 37 °C. When temperature condition of the microscopic chamber was set at 35 °C, in the untreated controls, the fluorescence ratio gradually decreased with time, reaching a plateau at the end of the protocol (mean fluorescence ratio: 0.505 ± 0.012 at 0 minute; 0.496 ± 0.013 at 60 minutes, P < 0.02) (Supplementary Table). This is probably because of the delayed response of the intracellular temperature to a decrease in the culture medium temperature that occurred when the culture dish was transferred from the incubator to the microscopic chamber. In contrast, the fluorescence ratio showed a marked increase with time following ANP (10^−7^ M) treatment (mean fluorescence ratio: 0.485 ± 0.012 at 0 minute; 0.491 ± 0.011 at 60 minutes, P < 0.03) (Supplementary Table), and CL316,243 (0.5 µM), a β3-adrenergic receptor (AR) agonist, treatment (mean fluorescence ratio: 0.507 ± 0.0084 at 0 minute; 0.513 ± 0.0080 at 60 minutes, P < 0.02). Furthermore, treatment with ANP (10^−9^ M) or isoproterenol (10^−7^ M) did not reduce the fluorescence ratio during the experimental protocol (mean fluorescence ratio: (ANP) 0.500 ± 0.015 at 0 minute; 0.503 ± 0.015 at 60 minutes, NS, (isoproterenol) 0.495 ± 0.009 at 0 minute; 0.501 ± 0.009 at 60 minutes, NS). The time course of the temperature-dependent changes in the fluorescence ratio (indicated as ∆fluorescence ratio) is shown in Fig. 4a. After 60 minutes of treatment with the indicated peptides/hormone, the ∆fluorescence ratios of the ANP-treated (10^−7^ M), isoproterenol-treated, and CL316,243-treated groups were significantly higher in comparison to the controls. Treatment with a more physiological dose of ANP (10^−9^ M) also tended to increase the ∆fluorescence ratio in comparison to the controls (P = 0.097). These data suggest the warming effects of ANP as well as β-AR agonists on brown adipocytes. On the other hand, the time course of the temperature-dependent changes in ∆fluorescence ratio at 37 °C is shown in Fig. 4b. In contrast to the data at 35 °C described above, ∆fluorescence ratios in each group were not significantly different compared to the untreated control group during the experimental protocol, although treatment with CL316,243 (0.5 µM) showed a tendency toward increase in the ∆fluorescence ratio in comparison to the controls (P = 0.061).

### ANP induces UCP1 transcription in brown adipocytes

**Figure 5:**
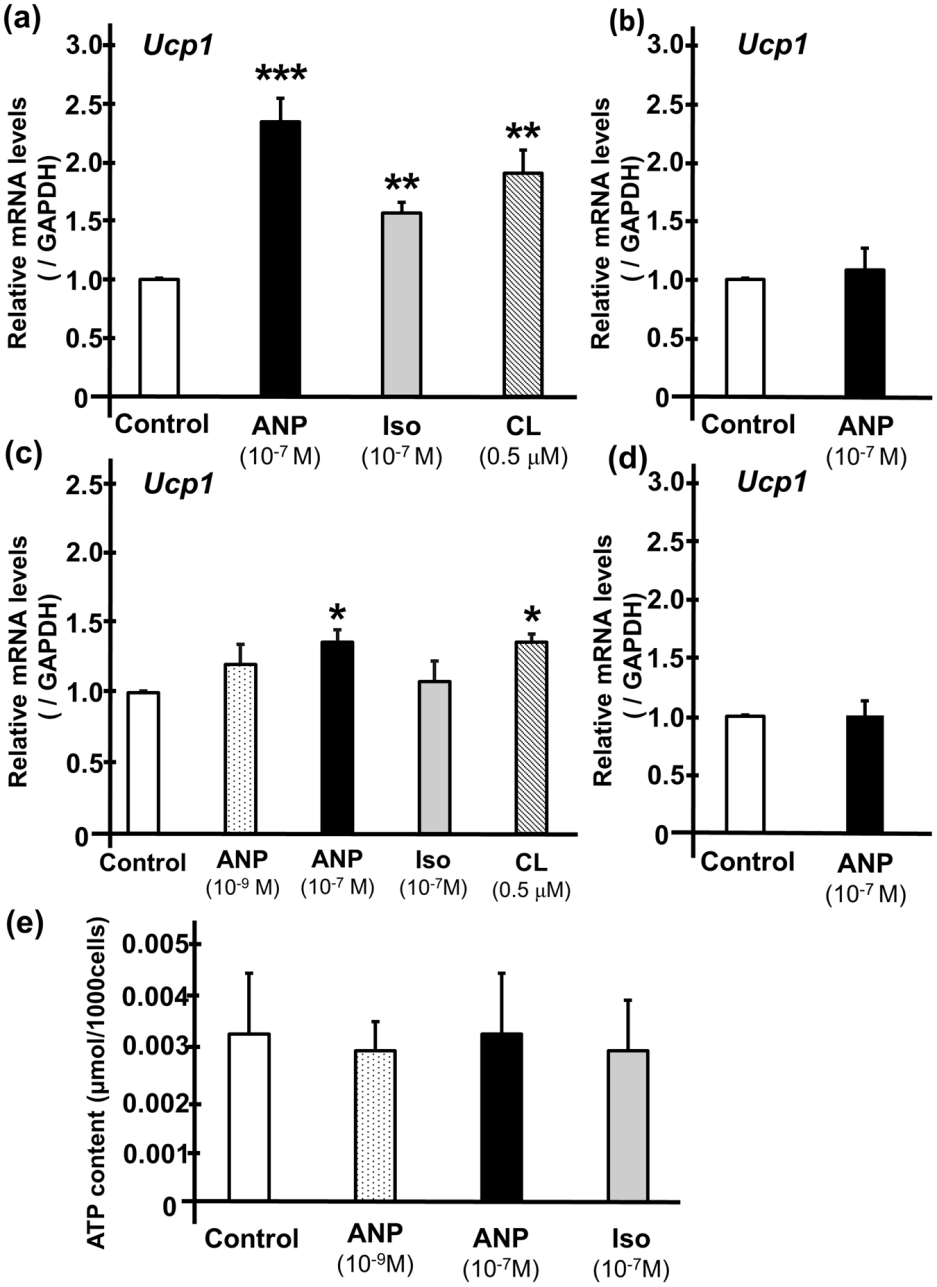
ANP increases the UCP1 levels in rat brown adipocytes. The quantification of the Ucp1 gene expression levels in rat brown adipocytes (day 7 or 8) after one hour of incubation at 35 °C with ANP (10^−7^ M), isoproterenol (10^−7^ M), or CL316,243 (0.5 µM) (n = 3 each) (**a**) and those after 30 minutes of incubation at 35 °C with ANP (10^−7^ M) (n = 3) (**b**) were shown. The *Ucp1* gene expression levels after one hour of incubation at 37 °C with ANP (10^−9^ M or 10^−7^ M, n = 4), isoproterenol (10^−7^ M, n = 4), or CL316,243 (0.5 µM) (n = 3) (**c**) and those after 30 minutes of incubation at 37 °C with ANP (10^−7^ M) (n = 3) (**d**) were also shown. The qPCR data were normalized to GAPDH. The data are shown as the fold change normalized to the levels found in untreated cells (control) *P < 0.05, **P < 0.03, ***P < 0.01 versus control (unpaired two-tailed Student’s t-test). (**e**) The ATP content in the adipocytes was measured after one hour of incubation with either ANP or isoproterenol (n = 3). Iso, isoproterenol, CL, CL316,243.

To investigate the possible mechanisms underlying the thermogenic actions of ANP, we examined the effects of ANP on the *UCP1* mRNA level. After one hour of treatment with either ANP (10^−7^ M), isoproterenol (10^−7^ M), or CL316,243 (0.5 µM) at 35 °C, the *UCP1* mRNA levels were significantly increased in comparison to the untreated control (Fig. 5a), which were more prominent than those at 37 °C (Fig. 5c). The increase in the *UCP1* mRNA level was more prominent after six hours of treatment with either ANP (10^−7^ M) or isoproterenol at 37 °C (Supplementary Fig. S3). There was no significant increase in the *UCP1* mRNA level after 30 minutes of treatment with ANP (Figs 5b,d), a time point at which the ANP-induced intracellular temperature increase did not reach statistical significance (Fig. 4). These data indicate that intracellular temperature increased in parallel with the induction of the UCP1 expression. On the other hand, one hour of treatment with ANP or isoproterenol did not affect the ATP content in brown adipocytes (Fig. 5e). These data suggest that ANP, rather than ATP synthesis, at least partly induces mitochondrial respiratory uncoupling through UCP1, which leads to heat generation in brown adipose tissue.

### The effects of ANP on the intracellular temperature and UCP1 transcription in white adipocytes

The changes in the intracellular temperature were also measured in white adipocytes. The time course of the ∆fluorescence ratio is shown in Supplementary Fig. S4. There was no significant increase in the intracellular temperature of white adipocytes after one hour of treatment with ANP, isoproterenol, or CL316,243 (Supplementary Fig. S4). Accordingly, the *UCP1* mRNA levels were not significantly increased after one hour of treatment with ANP, isoproterenol, or CL316,243 (Supplementary Fig. S5). These data suggest that short-term treatment with ANP does not exert significant thermogenic effects on white adipose tissue (WAT). However, given that the thermogenic program can be activated in WAT by stimulation with natriuretic peptides (NPs), through “browning”, which—in general—takes at least a number of hours, further studies with longer periods of ANP treatment are warranted to fully delineate the thermogenic actions of NPs in WAT.

### The role of the p38MAPK-UCP1 pathway in the thermogenic signaling cascade of ANP in rat brown adipocytes

**Figure 6:**
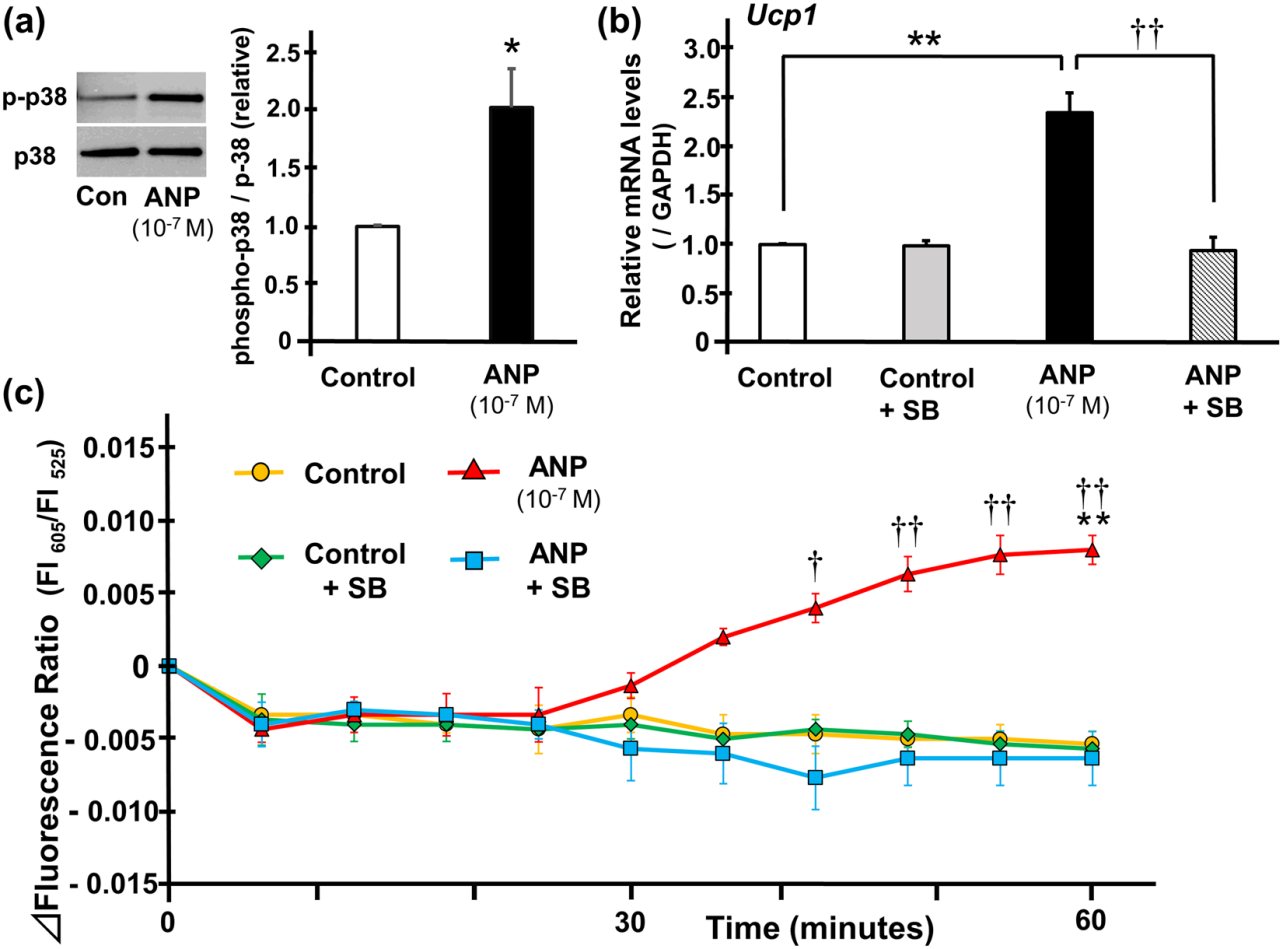
Thermogenic actions of ANP in rat brown adipocytes through p38MAPK-UCP1 pathway. (**a**) Phosphorylation of p38MAPK was evaluated in rat brown adipocytes treated with or without ANP (10^−7^ M) for one hour. Representative immunoblots obtained using the indicated antibodies are shown and full-length blots are presented in Supplementary Fig. S6. Averaged densitometry data normalized to the control at the same time points are shown in the bar graphs (n = 3). *P < 0.05 versus control. (**b**) The quantification of the *Ucp1* gene expression levels in rat brown adipocytes (day 7 or 8) after one hour of incubation at 35 °C with or without ANP (10^−7^ M) stimulated with either SB203580 or DMSO (n = 3) are shown. The qPCR data were normalized to GAPDH. The data are shown as the fold change normalized to the levels found in untreated cells (control). **P < 0.01 versus control; ^††^P < 0.01 versus ANP + SB. (**c**) The changes of the fluorescence ratio in rat brown adipocytes (day 8) after treatment with or without ANP (10^−7^ M) stimulated with either SB203580 or DMSO were recorded every 6 minutes at 35 °C (n = 3). The data represent the mean ± SEM. **P < 0.01 versus control; ^†^P < 0.03 and ^††^P < 0.01 versus ANP + SB at each time point (one-way ANOVA followed by Bonferroni’s multiple comparisons test). Con, control, SB, SB203580.

In order to elucidate the mechanism in greater detail, we investigated the role of the p38MAPK-UCP1 pathway in the possible thermogenic signaling cascade of ANP. First, we confirmed that the phosphorylation of p38MAPK was significantly increased after one hour of treatment with ANP (10^−7^ M) (Fig. 6a). Next, we investigated the effects of p38MAPK activation on UCP1 expression in BAT. The expression of UCP1, which was increased after one hour of treatment with ANP, was cancelled by the inhibition of p38MAPK with SB203580 (Fig. 6b). The time course of the ∆fluorescence ratio using a p38MAPK inhibitor is shown in Fig. 6c. As a result, the increase in intracellular temperature that was induced by one hour of treatment with ANP was canceled by SB203580 (Fig. 6c). These data suggest that ANP induces thermogenic actions on BAT through the activation of the p38MAPK-UCP1 pathway.

## Discussion

In the present study, we established a system to directly measure the intracellular temperature using a fluorescent thermoprobe under a fluorescence microscope. Using this novel system to directly measure the intracellular temperature, we demonstrated that ANP exerts warming effects on brown adipocytes. To assess the temperature change in greater detail, we developed an approximation formula based on the calibration (coefficient = 0.0074) (Fig. 3), and estimated the values more precisely, as follows: ANP (10^−9^ M); +1.55 °C, ANP (10^−7^ M); +2.18 °C, isoproterenol (10^−7^ M); +1.94 °C, CL316,243 (0.5 μM); +1.98 °C. Since the data collection for the calibration curve and the determination of the actual fluorescence intensity ratio is technically difficult to perform using the same microscopic system, these values remain speculative. However, because the calibration curve showed a proportional straight line in the measured range, we believe that those estimated values reflect the actual minute temperature change. Although it was previously reported the thermogenic action of the β-AR-cAMP pathway (which can be activated by isoproterenol) in brown adipocytes^14^, to our knowledge this is the first report to reveal the similar thermogenic effects of NPs on adipocytes. This represents a new insight into the actions of this cardiac neurohumoral factor.

Previous studies indicated the loss of heat in severe heart failure^19–21^. Nohria and Stevenson *et al*. showed that patients with heart failure in association with clinical hypoperfusion due to an impaired cardiac output manifest a “Cold” profile^19,20^. Reduced tissue perfusion is largely caused by the compensatory activation of the sympathetic nervous system and the renin-angiotensin-aldosterone system, which lead to a reduction in cardiac output in patients with heart failure. Moreover, Payvar et al. recently demonstrated that a low body temperature is associated with worse outcomes in patients with worsening heart failure^21^. A series of studies on soothing thermal/warmth therapy, called Waon therapy, showed beneficial effects in patients with heart failure^22–25^. Waon therapy induces thermal vasodilation, reduces the cardiac preload and afterload, and improves hemodynamics in subjects with heart failure. It has also been shown to reduce sympathetic nervous activity and oxidative stress, and to improve the vascular endothelial function through increases in endothelial nitric oxide (NO) synthase and NO production, all of which result in an improvement of the cardiac function, cardiac event rate and clinical symptoms. In this context, the thermogenic actions of ANP, as well as the soothing thermal therapy, could provide counter regulatory therapeutic effects against severe heart failure, although there might be other multiple cardioprotective mechanisms by keeping the heart warm.

Classical BAT, exemplified by the interscapular depots of rodents, is originally thought to have evolved as a compensatory defense system against hypothermia in mammals^26^. In a cold environment, the interscapular BAT depots show far greater thermogenic capacity than inguinal white adipose tissue (WAT), which occupies most of the WAT volume in mice^27^. The epicardial fat, located within the heart, has been suggested to function similarly to BAT and thus, could provide heat directly to the myocardium^28–31^. Although the present study clearly demonstrated the warming effects of ANP on brown adipocytes, accumulating evidence supports that a similar thermogenic program can be activated in the WAT by ANP stimulation, through “browning”, which leads to the formation of beige adipocytes^9,15^. These findings suggest the following compensatory adaptive mechanisms: the failing heart may protect itself against a decrease in hypoperfusion-induced temperature by producing NPs and utilizing their thermogenic actions in the fat tissue surrounding the myocardium.

Intriguingly, the increase in the intracellular temperature, as well as the increase in the expression of UCP1 that was induced by one hour of treatment with either ANP or β-AR agonists were more prominent at 35 °C than at 37 °C (Figs 4 and 5). These data indicate that the thermogenic actions of ANP on BAT are sensitive to low temperature, which makes sense when considering the clinical implications of the effects of NPs under conditions of severe heart failure in association with tissue hypoperfusion, as described above. Further studies are required to elucidate the mechanism underlying the low-temperature-sensitive warming effects of NPs.

A recent study by Bordicchia *et al*.^15^ revealed a novel mechanism through which NPs regulate the adipocyte function in a manner similar to that of catecholamines. Both NPs and catecholamines stimulate parallel pathways involving PKG and PKA, respectively, and promote lipolysis. Meanwhile, these two signals converge on p38MAPK activation, which leads to turning on the thermogenic program in brown and beige adipose tissues through the mitochondrial biogenesis induced by the elevated expression of PGC1α and UCP1^8,9^. In this context, UCP1, which is a BAT- and beige fat-specific protein, plays a pivotal role in heat generation by uncoupling mitochondrial respiration and ATP synthesis. Consistent with this theory, we found that the UCP1 level was increased in a p38MAPK dependent manner after treatment with a relatively high dose of ANP for one hour, whereas no significant change was observed in the cellular ATP content. Since the expression of UCP1 in BAT is naturally high, it is possible that either ANP or β-AR agonists increased UCP1 activity rather than the protein expression level itself. Further studies are warranted to fully delineate the role of UCP1 in the current model; the use of a UCP1 mRNA silencing system might be useful in this regard. Alternatively, using brown adipocytes from the UCP1 knockout mice would be a good experiment to perform. Meanwhile, it would be interesting to explore the precise mechanisms, other than p38MAPK, that regulate the UCP1 activation, such as ASK1 signaling^14^ and/or the IRE1α-XBP1 pathway^32^.

In order to more fully delineate the detailed cascades and the mechanisms of the thermogenic actions of ANP (namely, not only a downstream signaling cascade through p38MAPK-UCP1 pathway, but also an upstream receptor regulation), further investigation is required using NPR-A and/or clearance receptor knockdown system. It should also be noted that there might be dose-dependent differences in the effects of ANP after binding to NPR-A; specifically, low ANP concentrations induced adipogenic differentiation of atrial epicardium-derived cells via the cGMP-PKG pathways, whereas higher concentrations of NP induced lipolysis via cAMP-PKA pathways^33^.

It was reported that the expression of NP clearance receptors in rodents is approximately 100-fold greater than that in humans, and that this reduces NPR-A signaling^15^. This might be part of the reason why the significant thermogenic response of the brown adipocytes was observed with relatively high-dose ANP stimulation in the present study. On the other hand, previous studies showed that during fasting conditions, the expression of NPR-A was upregulated, while the expression of NP clearance receptors was downregulated^5,16^. Given that the rat brown adipocytes used in the present study were incubated in serum-free medium (namely, fasting conditions), ANP, when applied at a high dose, could still have a significant impact, even on these rodent-derived cells. These regulatory mechanisms of NP receptors gain in importance when considering the clinical implication. A recent study by Ryden *et al*.^11^ revealed that ANP-induced lipolysis is attenuated in obesity due to a decrease in NPR-A expression and an increase in NP clearance receptor expression. Therefore, it should be noted from a clinical standpoint that the thermogenic actions in response to ANP might be different in obesity subjects.

In the present study, the thermogenic action of ANP was not significant in WAT (Supplementary Figs S1,S2, S4,S5). In order to observe the thermogenic action of ANP in WAT, a technical issue needs to be solved. Specifically, a longer period of stimulation with ANP or β-AR agonists is likely to be required to induce WAT-browning (the transformation from white adipocytes to beige adipocytes generally takes more than a few hours); however, white adipocytes (as well as brown adipocytes) were intolerant of the unfavorable cytotoxic effects of the thermogenic probe that was used in the present study. Further studies are needed to clarify the warming effects of NPs on these beige adipocytes derived from WAT. The research is also important from a clinical standpoint, given that glucose metabolism is disturbed in heart failure (in which insulin resistance is highly prevalent)^34–36^, and UCP1 activation (in response to NPs) results in the improvement of glucose homeostasis in order to sustain high levels of uncoupled respiration within brown and beige adipocytes, which leads to the generation of heat^26^.

In summary, the thermogenic actions of ANP in brown adipocytes were directly proven using a novel intracellular temperature measurement system. NPs are produced and secreted in large amounts in the failing heart, and keep the heart warm through the thermogenic action in the epicardial fat (which possesses the features of BAT), and probably in the paracardial fat through browning. The present findings provide new insight into the self-protective role of NPs when the core body temperature or the local tissue (including heart itself) temperature fall due to unfavorable hemodynamic conditions in a state of severe heart failure.

## Methods

### Cell culture and differentiation

Rat brown adipocytes derived from the interscapular brown adipose tissue of adult SD rats were purchased from Cosmo Bio Co., and cultured using a Brown Adipocyte Culture Kit D-i (PMC-BAT10-COS) according to the manufacturer’s protocol. Rat brown preadipocytes were plated in 35 mm collagen-coated dishes (Sumitomo Bakelite Co.). The cells were cultured in growth medium for 4 days, then, in differentiation medium for 2 days, and in maintenance medium for 2 days. The differentiated brown adipocytes became confluent on day 7, and the maintenance medium was changed to serum-free medium for 18 hours. The cells were used in the following experimental protocol on day 8.

Rat white adipocytes derived from the subcutaneous white adipose tissue of adult SD rats were purchased from Cosmo Bio Co., and cultured using a White Adipocyte Culture Medium (PMC-SAC01-COS) according to the manufacturer’s protocol. Rat white preadipocytes were plated in 35 mm collagen-coated dishes, and cultured in differentiation medium. The medium was changed once in two days and the differentiated white adipocytes became confluent on day 7, and the differentiation medium was changed to serum-free medium for 18 hours. The cells were used in the following experimental protocol on day 8.

### The measurement of the intracellular temperature using fluorescent polymeric thermometers

The intracellular temperature in rat brown adipocytes was measured using Cellular Thermoprobe for Fluorescence Ratio (#FDV-0005, Funakoshi Co.) according to the manufacturer’s protocol. Rat brown and white adipocytes were prepared on a glass bottomed collagen-coated 35 mm dish, as described above. After washing twice with phosphate buffered saline (PBS), the cellular thermoprobe, which was dissolved in 5% glucose solution (0.05 w/v%), was added and the cells were incubated at 25 °C for 10 min. After washing twice (again) with PBS, 1 ml of phenol-red free Dulbecco’s modified eagle medium (DMEM) with L-glutamine was added and the cells were put back in the incubator at 37 °C with 5% CO_2_ until use. A Delta Vision (Airix Co.) fluorescence microscope was used to detect the thermoprobe in order to determine the fluorescence ratio. When measuring the fluorescence intensity, each dish was set up on the microscopic chamber at 35 °C or at 37 °C with 5% CO_2_. Where indicated, 20 µM of SB203580 (Tocris, Bristol, UK), p38MAPK inhibitor, was added to the medium at the beginning of stabilization period (Fig. 1). SB203580 was dissolved in dimethylsulfoxide (DMSO), and the solvent concentration was identically maintained in the control group. The cellular cytoplasm in which the thermoprobe was substantially introduced was selected and fixed under the microscope using a sampling square. The fluorescence intensity (FI) was measured every 6 minutes with excitation at 490 nm and dual emission at 525 nm and 605 nm, and the fluorescence ratio was calculated as FI 605 nm divided by FI 525 nm. After stabilization for 40 minutes, either 10^−9^ M or 10^−7^ M of ANP (carperitide, provided by Daiichi-Sankyo Pharmaceutical Co.) or 10^−7^ M of isoproterenol (Sigma-Aldrich) or 0.5 µM of CL316,243 (Tocris, Bristol, UK) (those were dissolved in distilled water, and the equivalent amount of distilled water was added to the control) was added directly to the indicated samples. The FI was subsequently measured every 6 minutes for 60 minutes. To evaluate the time-dependent change of the intracellular temperature, the ∆fluorescence ratio was calculated for each sample as the fluorescence ratio at the indicated time minus the ratio at 0 minutes (Fig. 1).

### The determination of the calibration curve for the fluorescent polymeric thermometers

Cell pellets of rat brown and white adipocytes were collected from 35 mm dishes and resuspended in hypertonic buffer (0.42 M KCl, 50 mM HEPES-KOH, 5 mM MgCl2, 0.1 mM EDTA, 20% glycerol, pH 7.8). The cells were then lysed using a 25-G needle with a syringe and centrifuged in order to collect the cell extract supernatant (11,000 r.p.m., 15 min, 4 °C). After diluting the supernatant to 40% with water (in order to adjust the KCl concentration to 0.15 M), 1 µl of the thermoprobe in water (1% w/v) was diluted with the cell extract supernatant (100 µl in total). The cell extract with the thermoprobe was loaded in a 96-well plate and the FI was measured by a plate reader equipped with temperature control (EnSpire, Perkin Elmer). The fluorescence ratio (excitation at 490 nm, emission ratio at FI 605 nm/FI 525 nm) was measured after the temperature of the plate chamber became steady (29–41 °C). The fluorescence ratio against the temperature was plotted to obtain the calibration curve.

### RNA isolation, Reverse Transcription (RT) and Real-Time Polymerase Chain Reaction (PCR)

After 30 minutes, 1 hour or 6 hours of stimulation by the indicated peptide/hormone (10^−7^ or 10^−9^ M of ANP, 10^−7^ M of isoproterenol, or 0.5 µM of CL316,243), each dish was snap frozen. Where indicated, 20 µM of SB203580 or equivalent volume of DMSO was added to the medium. Total RNA was extracted from the frozen cells using TRIzol reagent (Invitrogen) and a quantitative real-time PCR was performed using a StepOnePlus Real-time PCR System and the StepOne Software program (Applied Biosystems), as described previously^37^. The real-time PCR protocol consisted of one cycle at 95 °C for 20 s followed by 40 cycles at 95 °C for 1 s and 60 °C for 20 s using the primers for UCP1 (Applied Biosystems, Rn00562126_m1) and GAPDH (Applied Biosystems, Rn01775763_g1). The transcriptional levels were determined using the ∆∆Ct method with normalization to GAPDH.

### The measurement of the tissue ATP content

The ATP content in the rat brown adipocytes was determined using a firefly bioluminescence assay kit (AMERIC-ATP kit; Wako Pure Chemical Industries), as described previously^38^. Briefly, the dishes were washed twice with PBS, treated with 1 ml of trypsin and then harvested with 1 ml of high glucose DMEM with 10% bovine calf serum. After centrifugation (3500 r.p.m., 5 min, 4 °C), the supernatant was removed and the cell pellets were resuspended in saline water and centrifuged (3500 r.p.m., 5 min, 4 °C) twice. Then 100 μl of the sample was transferred into 1.5-ml microtubes containing 300 μl of manufacturer’s extraction liquid A (with phenol), 500 μl of extraction liquid B (with chloroform), and 500 μl of de-ionized water. The rest of the sample was used for cell counting by a hemocytometer. After being thoroughly shaken, the sample was centrifuged (10000 g, 5 min, 4 °C) in order to achieve phase separation, and 100 μl of the upper aqueous phase was stored at −80 °C. The measurement of the ATP levels was outsourced to Applied Medical Enzyme Research Institute Corporation (Tokushima, Japan).

### Immunoblotting

Immunoblotting was performed as previously described^38–40^ with rabbit monoclonal anti phospho-p38MAPK (1:1000, Cell Signaling #4511, Danvers, MA, USA), or mouse monoclonal anti p38MAPK (1:3000, BD Transduction Laboratories #612168, Franklin Lakes, NJ, USA). The signals were detected using chemiluminescence.

### Statistical analysis

The data are presented as the mean ± SEM of the indicated number of independent experiments. The statistical analyses included a one-way ANOVA followed by Bonferroni’s test for the correction of multiple comparisons; Student’s *t*-test was used for the comparison of two sets of data; two-way ANOVA was used for the comparison of the time-course analyses of Fluorescence Ratio profile (Supplementary Table). P values of < 0.05 were considered to indicate statistical significance.

## Electronic supplementary material

Supplementary Information
