# Supplementary material for: The thermogenic actions of natriuretic peptide in brown adipocytes: The direct measurement of the intracellular temperature using a fluorescent thermoprobe
Source: Sci Rep. 2017 Oct 11;7:12978. doi: 10.1038/s41598-017-13563-1 (PMC5636787; doi:10.1038/s41598-017-13563-1)
Supplement: Supplementary file 1 — Supplementary Information [file 41598_2017_13563_MOESM1_ESM.pdf]

Supplementary Information

The thermogenic actions of natriuretic peptide in brown adipocytes: The direct measurement of the intracellular temperature using a fluorescent thermoprobe

Haruka Kimura, Tomohisa Nagoshi*, Akira Yoshii, Yusuke Kashiwagi, Yoshiro Tanaka, Keiichi Ito, Takuya Yoshino, Toshikazu D. Tanaka, Michihiro Yoshimura

Supplementary Table. Fluorescence Ratio at each time point after treatment with ANP

Control (n)	0	54	60	(minute)
1	0.510±0.0199	0.491±0.0215	0.494±0.0235	
2	0.468±0.0174	0.457±0.0172	0.456±0.0174	
3	0.501±0.0159	0.487±0.0153	0.487±0.0157	
4	0.555±0.0171	0.549±0.0183	0.549±0.0190	
5	0.510±0.0285	0.509±0.0245	0.509±0.0249	
6	0.486±0.0142	0.483±0.0144	0.483±0.0143	
**ANP (n)**	**0**	**54**	**60**	**(minute)**
1	0.486±0.0189	0.497±0.0214	0.500±0.0215	
2	0.443±0.0147	0.449±0.0226	0.450±0.0220	
3	0.460±0.0156	0.462±0.0173	0.463±0.0180	
4	0.505±0.0201	0.513±0.0175	0.516±0.0183	
5	0.513±0.0204	0.519±0.0218	0.520±0.0222	
6	0.499±0.0163	0.499±0.0221	0.502±0.0270	

p=0.016, two-way ANOVA

Supplementary Figure Legend

Supplementary Figure S1. Representative microscopic images of rat white adipocytes treated with the fluorescent polymeric thermometer. A differential interference contrast image **(a)**, a fluorescence image (490 nm excitation, 525 nm emission), **(b)** and a fluorescence image (490 nm excitation, 605 nm emission) **(c)** of the cellular thermoprobe in rat brown adipocytes on day 8. A merged image of (b) and (c) with the sampling square of the measurement are also shown in **(d)**. Scale bar: 40 µm.

Supplementary Figure S2. The calibration curve of the fluorescent polymeric thermometer in rat white adipocytes. The responses of the fluorescence ratio (605nm/525nm) were analyzed (n=4). The data indicate the mean ± SEM.

Supplementary Figure S3. ANP increases the UCP1 levels in rat brown adipocytes. The quantification of the *Ucp1* gene expression levels in rat brown adipocytes (day 7 or 8) after six hours of incubation with ANP (10^{-9} M or 10^{-7} M) or isoproterenol (10^{-7} M) (n=5) at 37°C. The qPCR data were normalized to GAPDH. The data are shown as the fold change normalized to the levels found in untreated cells (control) *$P < 0.05$ versus control (unpaired two-tailed Student's t-test). Iso, isoproterenol.

Supplementary Figure S4. The profile of the intracellular temperature change in rat white adipocytes incubated with ANP. The intracellular temperature was indicated by the fluorescence ratio (605 nm/525 nm). The changes of the fluorescence ratio in rat white adipocytes (day 8) after treatment with ANP (10^{-7}M), isoproterenol (10^{-7}M), or CL316,243 (0.5 µM) were recorded every 6 minutes at 35°C (n=3 each). The data represent the mean ± SEM.

Supplementary Figure S5. ANP does not increase the UCP1 levels in rat white adipocytes. The quantification of the *Ucp1* gene expression levels in rat white adipocytes (day 7 or 8) after one hour of incubation with ANP (10^{-7} M), isoproterenol (10^{-7} M), or CL316,243 (0.5 µM) (n=3 each). The qPCR data were normalized to GAPDH. The data are shown as the fold change normalized to the levels found in untreated cells (control) Iso, isoproterenol. CL, CL 316,243.

Supplementary Figure S6. Full-length blots of phospho-p38 and total p38.
Western blotting of phosphorylation of p38MAPK and total p38MAPK in rat brown adipocytes treated with or without ANP (10^{-7} M) for 60 minutes. M, MagicMark XP Western Protein standard, a molecular weight marker. kDa, kilo Dalton.

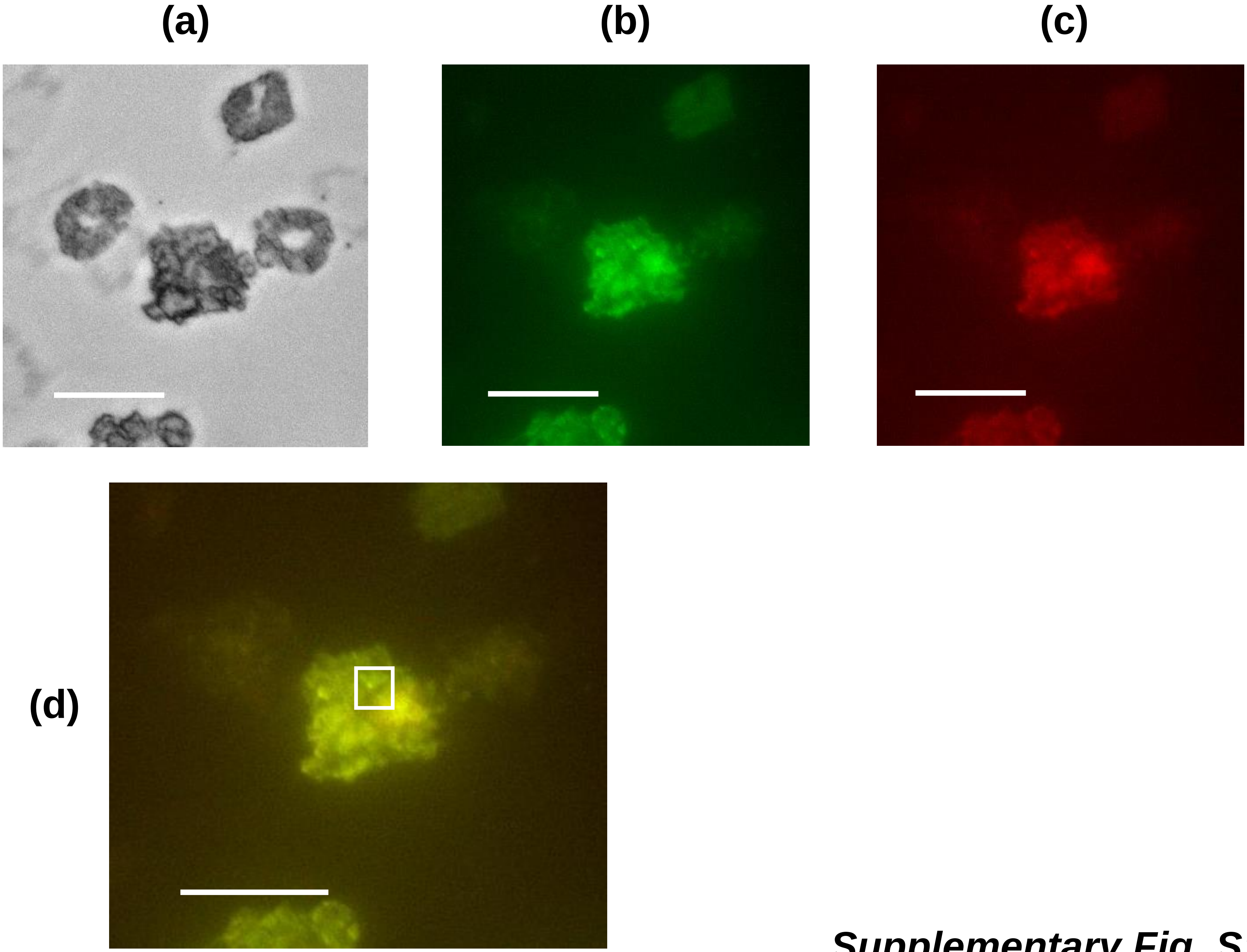

Supplementary Fig. S1

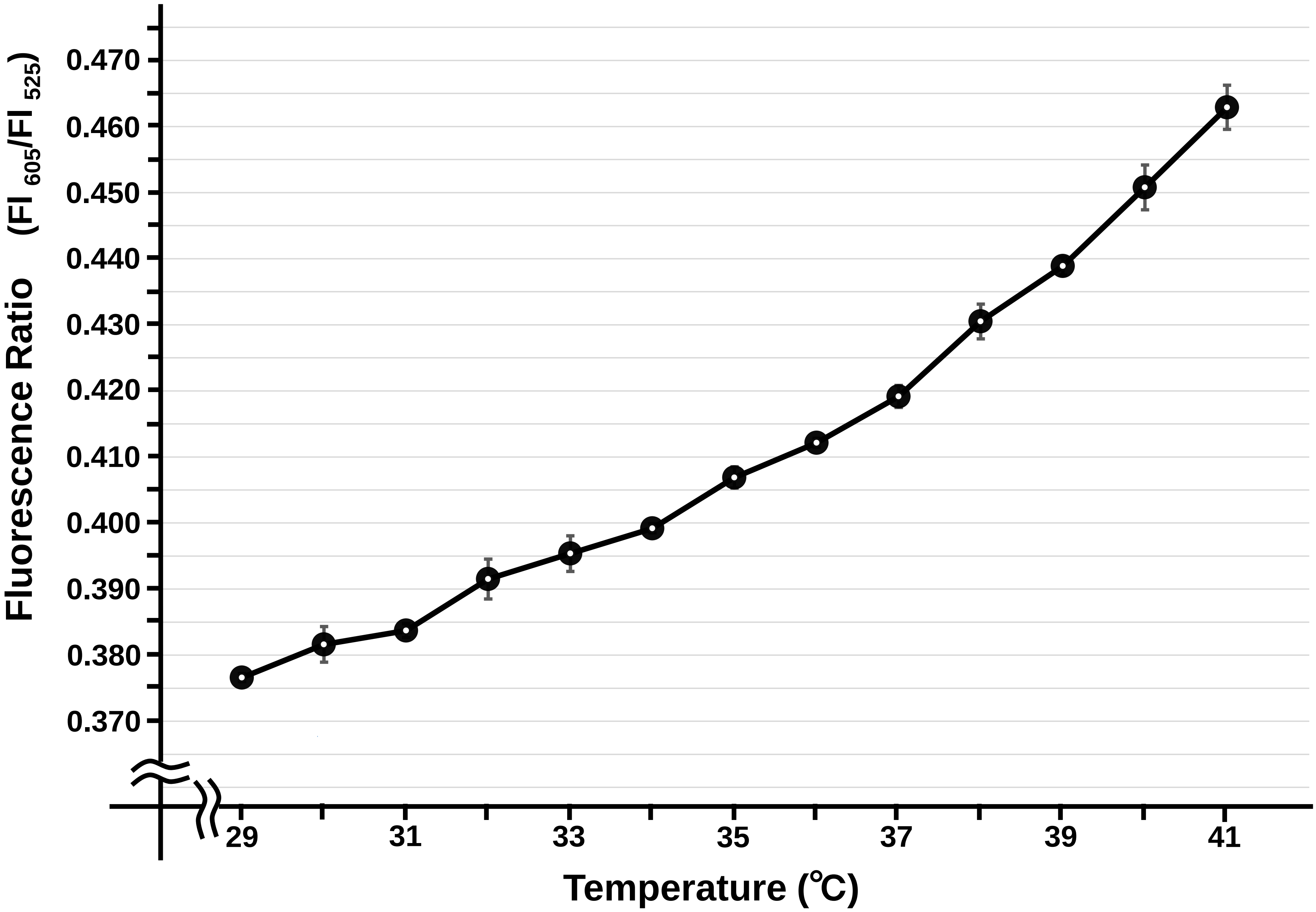

Supplementary Fig. S2

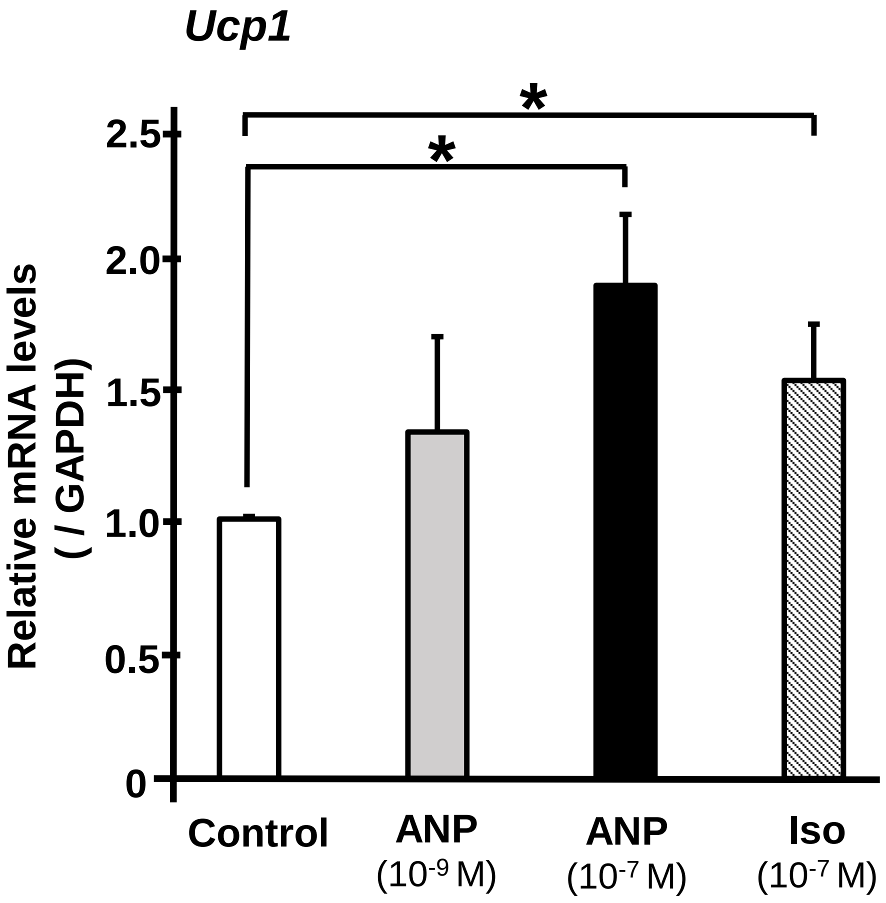

Supplementary Fig. S3

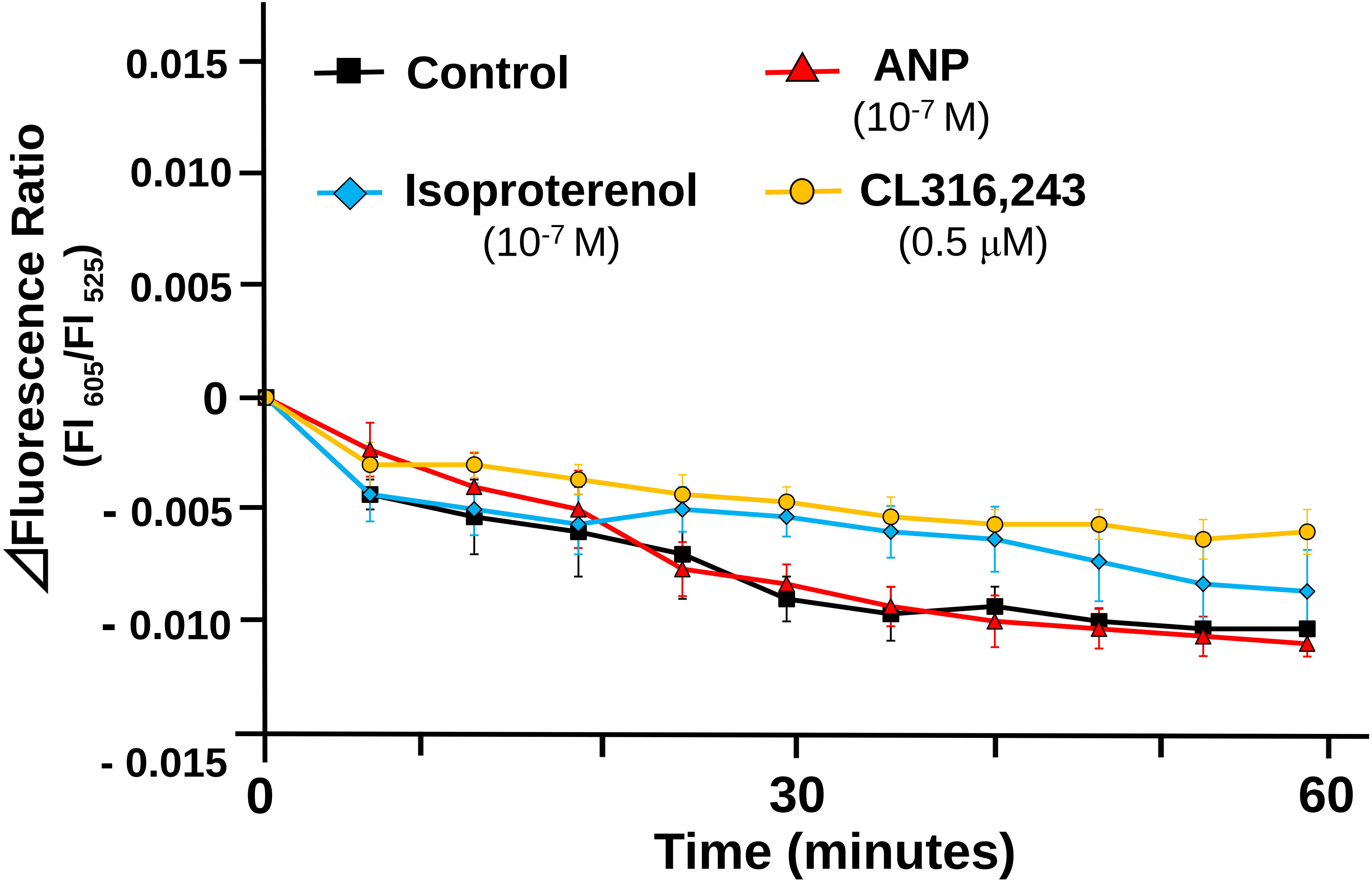

Supplementary Fig. S4

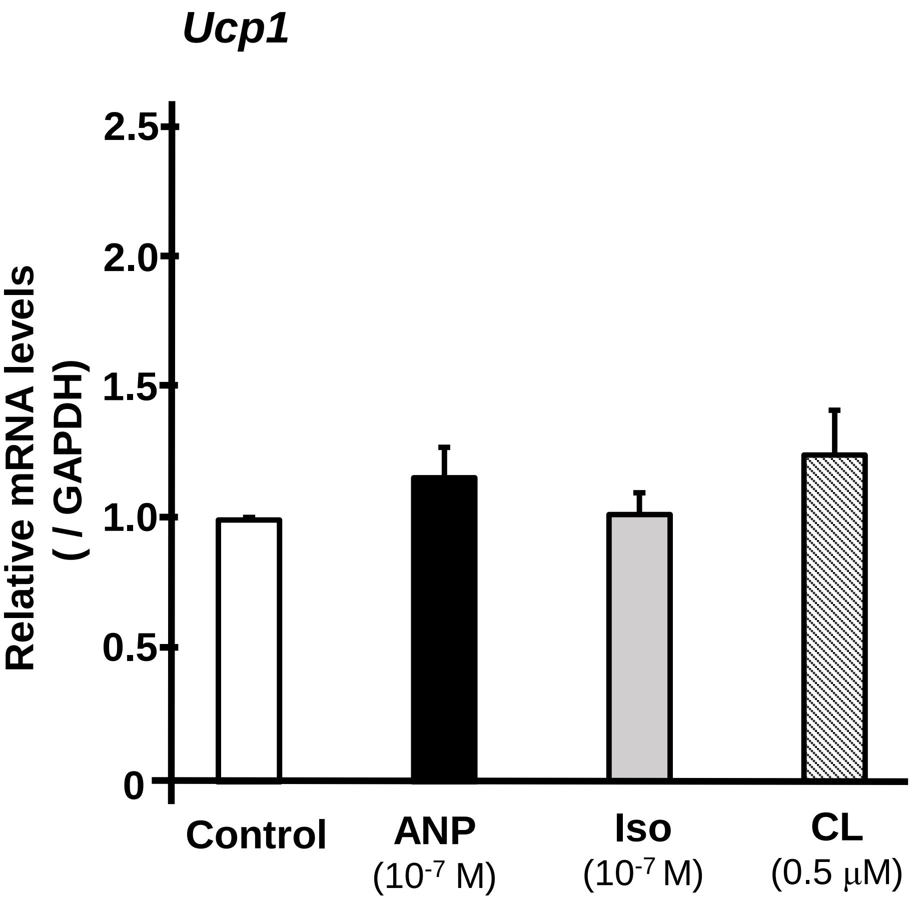

Supplementary Fig. S5

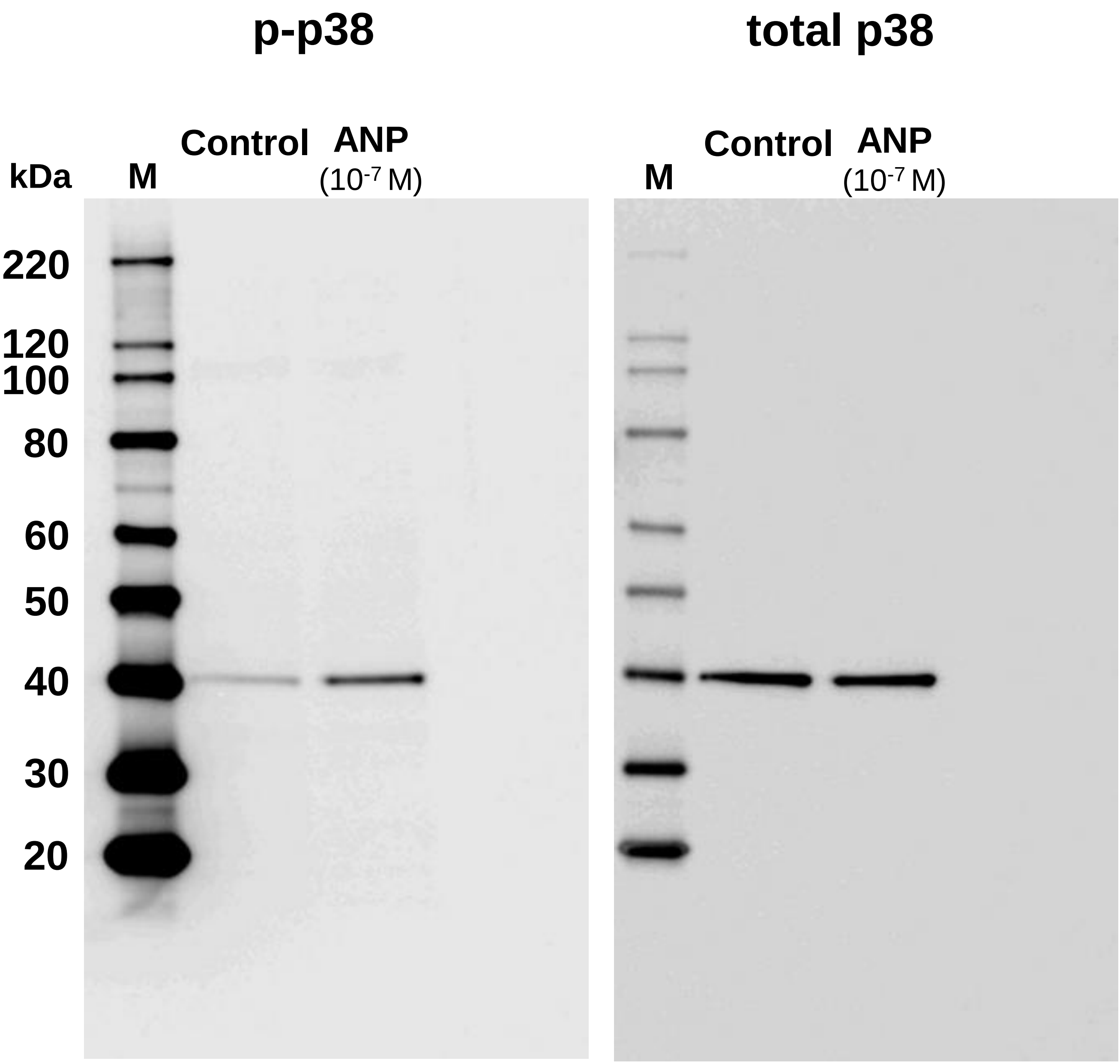

Supplementary Fig. S6
